# Management of Endometrial Cancer: French Society of Onco-Gynecology‘s Evaluation through a Delphi Survey

**DOI:** 10.3390/jcm11226765

**Published:** 2022-11-15

**Authors:** Carolin Marti, Elise Deluche, Floriane Jochum, Sofiane Bendifallah, Henri Azais, Jonas Deidier, Vincent Cockenpot, Inès Menoux, Vincent Balaya, Sarah Betrian, Cyrus Chargari, Sébastien Gouy, Catherine Genestie, Anis Feki, Catherine Uzan, Frederic Guyon, Mojgan Devouassoux-Shisheboran, Noémie Body, Cherif Akladios, Patrice Mathevet, Benedetta Guani, on behalf of the SFOG and the SFOG Campus

**Affiliations:** 1 Faculty of Biology and Medicine, University of Lausanne (UNIL), 1015 Lausanne, Switzerland; 2 Department of Gynecology and Obstetrics, HFR Fribourg-Hôpital Cantonal, 1708 Fribourg, Switzerland; 3 Department of Medical Oncology, CHU Limoges, 87000 Limoges, France; 4 Residual Tumor & Response to Treatment Laboratory, RT2Lab, Translational Research Department, INSERM, U932 Immunity and Cancer, University Paris, 75005 Paris, France; 5 Department of Gynecology and Obstetrics, Hopitaux Universitaires de Strasbourg, 67091 Strasbourg, France; 6 Department of Gynecology, Tenon Hospital, 75020 Paris, France; 7 Gynecologic and Breast Oncologic Surgery Department, Georges Pompidou European Hospital, 75015 Paris, France; 8 Department of Radiology, Hôpital Universitaire Paris Ouest Site G Pompidou APHP, 75015 Paris, France; 9 Department of Pathology, Centre Léon Bérard, 69008 Lyon, France; 10 Department of Radiotherapy, ICANS-Strasbourg-Europe Cancer Institute, 67200 Strasbourg, France; 11 Department of Gynecology, Foch Hospital, 92150 Suresnes, France; 12 Department of Medical Oncology, IUCT Oncopole, 31059 Toulouse, France; 13 Department of Radiation Oncology, Gustave Roussy Cancer Campus, 94850 Villejuif, France; 14 Department of Surgical Gynecology Oncology, Gustave Roussy Cancer Campus, 94850 Villejuif, France; 15 Department of Pathology, Gustave Roussy Cancer Campus, 94850 Villejuif, France; 16 Faculty of Science and Medicine, University of Fribourg (UNIFR), 1700 Fribourg, Switzerland; 17 Departement of Breast and Gynecologic Surgery, AP–HP, Hôpital de la Pitié Salpêtrière, 75013 Paris, France; 18 Institut Universitaire de Cancérologie, Sorbonne Université, 75006 Paris, France; 19 Department of Surgical Oncology, Bergonié Institute, 33076 Bordeaux, France; 20 Department of Pathology, Hospices Civiles de Lyon, 69002 Lyon, France; 21 Department of Surgical Oncology, Institut de Cancérologie de l’Ouest (ICO), 49055 Angers, France; 22 Department of Gynecology and Obstetrics, Lausanne University Hospital (CHUV), 1011 Lausanne, Switzerland

**Keywords:** endometrial cancer, Delphi procedure, SFOG guidelines, endometrial cancer guidelines

## Abstract

Our aim was to assess the opinion of a panel of experts and obtain a consensus on the management of endometrial cancer in France and French Switzerland. A Delphi survey was carried out among a panel of French and French-speaking Swiss experts. The first questionnaire included 65 questions divided into eight categories: characterization of experts, histo-molecular characteristics and radiological data of endometrial cancer, and management of low-risk, intermediate-risk, intermediate–high-risk, high-risk, and metastatic cancers. The experts were asked to reply on a 9-point scale, both on the validity and the clarity of each question. After the answers were analyzed, a second questionnaire was sent to the same experts. The study took place between December 2021 and March 2022. Further, 58 (57.4%) of the 101 experts responded in the first round, and 39 recommendations were obtained (60%). Six questions were voted redundant and 20 discordant. These questions were reformulated, and, at the end of the second round, 17 recommendations were validated (85%). In total, the study presents an analysis of 56 questions and related responses. Expert advice helps to clarify non-consensual issues, standardize the management of endometrial cancer, and optimize clinical practices.

## 1. Introduction

Endometrial cancer is the fourth most frequently diagnosed cancer in Europe, with 73,333 new cases and 16,773 deaths in 2020. Worldwide, endometrial cancer ranks sixth, with 417,000 new cases and 97,000 deaths in 2020 worldwide [1]. The average age at diagnosis is 68 years [2].

The incidence has increased with ageing and rising obesity levels in the population, but the number of deaths remains stable [3].

The first European consensus conference on endometrial carcinoma in 2014 defined multidisciplinary evidence-based recommendations [4,5].

The European Society of Gynecological Oncology (ESGO), the European Society of Radiotherapy and Oncology (ESTRO), and the European Society of Pathology (ESP) have recently updated their recommendations [5]. These guidelines leave some unanswered questions, and some answers are not agreed upon. To clarify these points, in collaboration with the French Society of Onco-Gynecology (SFOG) and the Young Members of SFOG (SFOG Campus), we decided to organize a Delphi and provide a framework for the development of a consensus in France and French Switzerland.

Our main goal was to evaluate the opinion of a panel of experts on the following topics: radiology, pathology, the role of the sentinel lymph node, fertility preservation, and management of low, intermediate, intermediate–high, high-risk, and metastatic tumors.

## 2. Materials and Methods

The Delphi method was originally designed in the 1950s by Olaf Helmer and Norman Dalkey of the Rand Corporation for the United States Army [6]. The Delphi method allows experts to work toward a mutual agreement by responding to a series of questionnaires and sharing the corresponding feedback to advance the discussion in each subsequent round. The experts’ responses evolve over the course of the rounds based on the information provided by the other experts participating in the analysis [7].

It is a method of consensual choice used in medical research to obtain the opinions of experts in a particular field [8]. We used a modified Delphi method with two rounds of questionnaires and two videoconferences to review the results. The method for developing the critical review of the endometrial cancer guidelines consisted of three phases: preparatory, expert, and national consensus.

### 2.1. Preparatory Phase

The objective of the preparatory phase was to pre-select potential questions to be developed. This phase was carried out by SFOG Campus. First, a detailed reading of the ESGO-ESTRO-ESP recommendations was performed. The elements of the recommendations were then analyzed according to their level of evidence and the practical questions raised regarding their applicability in France. Using a reading grid, they were classified into several categories according to stage and risk (low-risk, intermediate-risk, intermediate–high-risk, high-risk, metastatic tumor), phase of care (diagnosis, treatment, follow-up), and management (imaging, surgery, oncology, radiotherapy).

### 2.2. Expertise Phase

The objective of the expert assessment was to find a consensus among the experts on a list of questions. A total of 101 national and French-speaking Swiss experts were invited to participate. The selection was intended to represent health professionals working with endometrial cancer patients. The panel was multidisciplinary, coming from both the public and private sectors. Each expert completed a declaration of interest, and no conflicts were presented. The questionnaire was completed online using the Google Forms platform. The list of questions is summarized in Table 1.

To be included in the final selection, an indicator must meet the following three conditions:

− panel median > 7 for validity
− panel median > 6 for relevance
− no disagreement within the panel

Disagreement is statistically defined when both of the following conditions are met:

− 1/3 or more of the ratings are between 1 and 3
− 1/3 or more of the ratings are between 7 and 9.

First round of voting: The experts were asked to answer the questions to the first questionnaire and rate each question’s relevance and validity.

The rating was completed individually, without consultation with the other experts, and by return mail.

Videoconferences: Two meetings in the form of videoconferences took place on 8 December 2021 and 5 January 2022. After a presentation of the results of the first round of scoring, the experts were able to discuss the results, the formulation of the wording of the indicators, and their rating in terms of relevance and validity. This exchange allowed the experts to justify their choice and find solutions for the points of disagreement.

**Figure 1:**
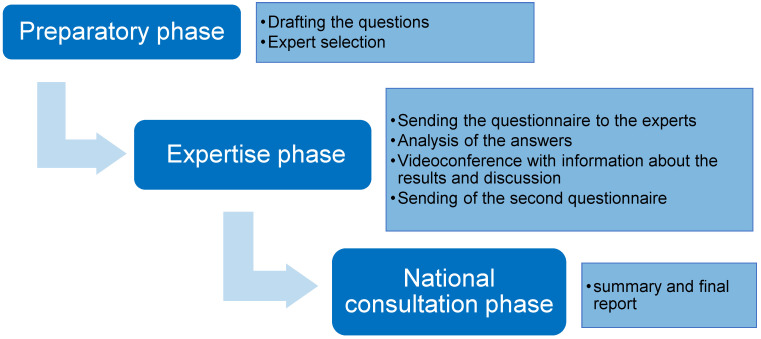
General organization of the procedure.

Second round of rating: The second round of ratings was carried out after the video conferences. The general organization of the procedure is shown in Figure 1.

## 3. Results

The study took place between December 2021 and March 2022. Further, 58 (57.4%) of the selected 101 experts belonging to the French Society of Onco-Gynecology responded in the first round, including twenty-six onco-gynecologists (44.8%), thirteen oncologists (22.4%), nine radiotherapists (15.5%), six anatomopathologists (10.3%), and four radiologists (6.9%). In the second round, 20 experts responded, i.e., 34.5% of the experts who also responded in the first round.

The 58 participants were predominantly men (56.1%), with a median age of 45 years (30–64 years) and 20 years of practice in their specialty (3–36 years).

The questionnaire consisted of 71 questions divided into eight groups: characterization of the experts, histo-molecular characteristics and radiological data for endometrial cancer, and the management of low-risk, intermediate-risk, intermediate–high-risk, high-risk, and metastatic cancer.

At the end of the first round, 39 of the 65 proposals (excluding six questions on expert characteristics) were accepted (60%). A total of six questions, three of radiotherapy, two of onco-gynecology, and one of radiology, were voted redundant and twenty discordant, sixteen of oncology, two of pathology, one of radiology, and one of radiotherapy. These 20 questions were reformulated and sent in a questionnaire for a second round. Twenty original experts (34.5%) participated in the second round, and, of the twenty questions, seventeen were validated and three were rejected (two questions of oncology and one of onco-gynecology). In total, the study presents an analysis of 56 questions and related responses.

The summary of all validated proposals is shown in Table 1. Some of the key items not specified in the ESGO-ESMO (European Society of Medical Oncology)-ESP Guidelines are presented below.

### 3.1. Fertility Preservation

If the patient decides to preserve fertility, the treatment of choice is an oral progesterone.

The Delphi showed that the experts support fertility preservation in patients with FIGO 1A grade 1 tumors as well as grade 2. If the fertility preservation route is chosen, a final hysterectomy should be suggested if pregnancy does not occur within two years.

### 3.2. Lymph Node Assessment

Inexperienced surgeons should perform at least 20 sentinel node biopsies before performing a biopsy without supervision. Indocyanine green is the preferred tracer, even outside clinical trials (off-label prescription). In the case of a hysterectomy, a uterine manipulator with a cannula cannot be used for the procedure.

Sentinel lymph node removal should be performed as a preventive measure even if there is no suspicion of infiltration of the myometrium on imaging.

The experts could not reach a consensus on the use of Persson’s algorithm but advocated its use. There is no consensus on the need for repeat lymph node dissection in the case of a positive pelvic sentinel node in the early stages, nor is there a consensus on the value of frozen section analysis of sentinel nodes.

### 3.3. Adjuvant Treatment

Adjuvant treatment recommendations for endometrial carcinoma depend strongly on the prognostic risk group.

#### 3.3.1. Intermediate Risk

Brachytherapy should be continued in patients with stage IA non-endometrioid tumors (i.e., serous type, clear cell undifferentiated carcinoma, carcinosarcoma, mixed carcinoma) without myometrial invasion. However, it may be omitted in patients aged less than 60 years.

#### 3.3.2. Intermediate–High Risk

Most experts (55.6%) suggest extended lombo-aortic radiotherapy if the PET scan is positive. The other options were systematic para-aortic-lymph node dissection (27.9%) and para-aortic-lymph node dissection if PET-negative (16.7%).

A sentinel lymph node procedure for lymph node staging in high-risk cancers to be treated with radiotherapy is recommended by most experts.

#### 3.3.3. High Risk

High-risk endometrial cancers should not be treated with chemotherapy alone, and closing surgery is recommended if there is a good response to chemotherapy and no comorbidity for surgery.

## 4. Discussion

The Delphi method has some limitations in that there is no evidence that Delphi studies are reliable (i.e., if two panels are provided the same question, they may not reach the same consensus), so the success of a Delphi study is highly dependent on the quality and experience of the expert panel.

The presence of consensus does not necessarily mean that the right answer or opinion or judgment has been found; it simply helps to identify the areas that a group of participants or experts consider important in relation to that topic. Another issue is that continuous engagement is required from participants who are asked a similar question multiple times, which may be one reason why experts drop out in later rounds of the study.

Although the Delphi method has some limitations, this study has allowed us to reach a consensus on various issues arising from the ESGO-ESTRO-ESP 2021 recommendations [5].

These guidelines have raised many questions among the experts in gynecologic oncology, especially regarding the introduction of molecular biology, which is not always available in all centers and is often only analyzed after surgery.

Furthermore, the European Society for Medical Oncology did not participate in the 2021 guidelines, resulting in a lack of information and consensus on the adjuvant treatment of endometrial cancer, and we were able to involve endometrial cancer experts from all disciplines, which leads to wider acceptance.

Our Delphi aims to respond to many points that remain unclear or are left to the discretion of the operator when reading the guidelines. Additionally, the French guidelines are still from 2010 and thus need to be renewed, and there are no national guidelines in Switzerland; the university centers in French-speaking Switzerland have common internal guidelines based on the ESGO guidelines. For these reasons, the SFOG and the SFOG Campus decided to create this Delphi to generate a consensus supported by the experience and knowledge of experts from France and French-speaking Switzerland. In our Delphi, we have included experts from all specialties confronted with this type of cancer, as well as radiologists and radiotherapists who did not participate in drafting the European Guidelines.

In the following discussion, we will outline some basic points.

### 4.1. Radiology

In the field of radiology, the experts’ agreement was very high: the experts emphasize the need to perform an MRI for all patients with a histological diagnosis of endometrial cancer. Therefore, the exam should preferably be performed after histological evidence. Ultrasound remains the first choice for detection of endometrial lesions but is inadequate for lymph node analysis. For this reason, in the case of inaccessibility to an MRI (magnetic resonance imaging), a CT scan (computerized tomography) is recommended in addition to a US (ultrasound) [9].

There is no consensus on performing a PET CT (positron emission tomography scan) in the workup for all endometrial cancers, only in high-risk cases. PET CT has an excellent specificity for the preoperative assessment of lymph node metastases, which explains why experts prefer the result of PET CT over MRI in cases of discrepancy on lymph node status [10,11].

### 4.2. Pathology

Regarding molecular analysis, many points of discussion were noted.

Since molecular analysis often alters prognosis and treatment, it was essential to reach a consensus and provide a recommendation for all French and French-speaking Swiss colleagues that could then be adopted in Europe.

Molecular analysis on a biopsy before surgery is difficult to access. However, most experts (83.3%) recommended characterizing the biopsy as completely as possible, and, if not, at least performing p53 and MMR-IHC.

The search for DNA polymerase epsilon (POLE) mutation is expensive and requires next-generation sequencing (NGS). The experts recommend that routine testing should not be generalized but that POLE testing be limited to cases where it can modify treatment.

- Unnecessary: Low-grade tumors without embolus stage IA p53 normal and Stages III/IV
- To be discussed on a case-by-case basis: In cases of non-endometrioid histology
- Necessary: Stages I/II, especially high-grade endometrioid

Concerning Lynch Syndrome, according to European recommendations, experts recommend (78.9%) a complete MLH1/PMS2/MSH2/MSH6 panel by immunohistochemistry. Obtaining endometrial sampling by biopsy or D&C (dilation and curettage) is an acceptable initial approach for histologic diagnosis of endometrial cancer [12]. If possible and if the material is available and sufficient in quantity and quality, it is advisable to perform the molecular analysis immediately after the biopsy to obtain all the elements useful for evaluation of the risk group and to better control the preanalytical analysis as soon as possible.

To identify patients with Lynch syndrome and triage them for germline mutational analysis, MMR-IHC (plus MLH1 promoter methylation status analysis in the case of immunohistochemical loss of MLH1/PMS2 expression) or MSI testing should be performed in all endometrial carcinomas, regardless of the histological subtype of the tumor (III, B).

This approach is widely available and cost-effective for identifying patients with a higher probability of having Lynch syndrome [5].

### 4.3. Lymph Node Status

Classically, the indication for lymph node staging depended on the preoperative risk group. However, lymph node dissection has no therapeutic impact on survival [13,14,15], even if it allows one to correctly classify the postoperative risk group and adapt the adjuvant treatment.

The sentinel lymph node has, therefore, been introduced as an alternative to lymph node dissection. If the procedure is performed according to the recommendations, a negative sentinel node is accepted to confirm the absence of positive lymph nodes (N0). The sentinel node procedure, applicable to all presumed early stages (I and II) regardless of histology and imaging, provides this information with less morbidity and avoids a possible pre- versus postoperative discrepancy in the risk group assessment [16,17,18,19]. While retrospective studies regarding survival after performing a sentinel node procedure have been published [20], prospective survival data are pending (SentiRAd).

In the guidelines, sentinel lymph node biopsy may be considered for staging purposes in low-risk or intermediate-risk patients without lymphadenectomy. However, it may be omitted in the absence of myometrial invasion. (II, A) [5].

Sentinel node is an alternative for the “high–intermediate” and “high” risk groups, regardless of histologic type.

The points still debated are the complement in the case of pelvic micro- or macro-metastasis (complementary lumbo-aortic dissection or radiological lymph node evaluation), the type of pathological ultra-staging protocol, and the relevance of the frozen section.

After two consultation rounds, we did not reach a consensus regarding complementary lumbo-aortic lymphadenectomy in cases of positive pelvic sentinel lymph nodes. However, the majority (60%) of our experts predicted surgical re-staging. New prospective data are needed to adjudicate this point.

Regarding the other points, experts do not consider frozen sections necessary since they only detect micro-metastases in very few cases. They also recommend ultrastaging analysis of the all-sentinel lymph nodes.

### 4.4. Adjuvant Treatment

When molecular classification is known, the presence of POLE mutation in patients with stages I–II justifies the omission of adjuvant therapy (III, A) [5]. In fact, the PORTEC-3 study showed that patients with endometrioid carcinoma and a POLE mutation had excellent prognoses [21,22].

In stages III–IV, the analysis for the POLE mutation is not indicated. The data in the literature are insufficient to justify the absence of adjuvant treatment (IV, C) [5]. A prospective database of POLE-mutated patients is strongly recommended to evaluate the treatment and prognosis of these patients.

In the case of p53 mutations, the prognosis for the patient appears to be poor: abnormal p53 tumors expression is categorized as follows: strong positive p53 expression in >80% of the tumor nuclei (mutant overexpression), complete absence of p53 expression with a positive internal control (null mutant), or significant cytoplasmic p53 expression (cytoplasmic) in >80% of the tumors. Subclonal abnormal p53 expression is defined as any abrupt and regional abnormal p53 expression in less than 80% of the tumor volume [23,24].

These p53abn carcinomas tumors are at high risk for recurrence in the case of myometrial invasion. The benefit of multimodal treatment is clear for p53abn carcinomas regardless of histologic subtype [21,22]. Stage I mutated p53 should be treated globally as the high-risk group, except for stage IA without endometrial invasion. In cases of p53abn tumors without myometrial invasion or tumors limited to a polyp, adjuvant treatment may be discussed regarding the lack of data in clinical trials.

### 4.5. Prospective

All the points discussed in the Delphi will be incorporated in the Franco-Swiss guidelines, which are being developed in collaboration between SFOG, SFOG Campus, and French-speaking experts from France and Switzerland.

We hope that this work will lead to stronger recommendations and facilitate a consensus.

## 5. Conclusions

These consensual recommendations should enable standardization of management of endometrial cancer in France and French-speaking Switzerland and optimize clinical practices. They attempt to answer most of the questions asked daily by any physician treating endometrial cancer.

Given the multidisciplinary nature of the treatment of patients with endometrial cancer, we advocate that e4ndometrial cancer should be treated in specialized centers, especially in high-risk or advanced-stage patients, and that cases should always be discussed by a multidisciplinary tumor board.

## Figures and Tables

**Table 1:** List of Delphi questions.

	Degree of Agreement of Experts (Positive Answers)	Validation in Round 1 or 2
Radiology		
What is the diagnostic value of a pelvic MRI for the diagnosis of endometrial cancer?	100.0%	Round 1
2. Do you confirm the need for proven histology before performing an MRI?	100.0%	Round 1
3. Should the indication of MRI be extended to all patients with a proven histology of endometrial cancer?	75.0%	Round 1
4. When the MRI staging is uncertain/non-contributory (pathological myometrium: myoma, adenomyosis, cornual localisation, etc.), should a maximalist attitude be adopted, giving preference to surgery?	100.0%	Round 1
5. Should a PET scan be performed in the work up of all endometrial cancers?	44.4%	Round 2
6. In case of discrepancies in MRI vs. PET/CT imaging on lymph node status, which result should be used for treatment?	75.0% in favour of PET	Round 1
7. What follow up images should be done after initial treatment for low-risk endometrial cancers?	-clinical follow-up: 52.6% -no follow-up: 26.3% -MRI: 10.3%	Round 2
8. What follow up images should be done after initial treatment for high-risk endometrial cancers?	-PET scan: 31.6% -Clinical follow-up: 21.1%	Round 2
Pathology		
9. Should molecular biology be performed on non-endometrial tumours?	83.3%	Round 1
10. Concerning the evaluation of MMR status in the context of molecular classification in the absence of Lynch, what do you recommend?	Performance of a complete MLH1/PMS2/MSH2/MSH6 panel in IHC and confirmation in molecular biology if loss of expression: 78.9%	Round 2
11. Regarding ultrastadification on pelvic sentinel lymph nodes	ultrastadification HES + IHC: 66.7%	Round 1
12. Should we routinely search for L1CAM?	16.7%	Round 1
Fertility preservation		
13. What should be the treatment of choice for fertility preservation?	Oral progestin: 63.6%	Round 1
14. Can fertility preservation in FIGO 1A patients be considered in grades 2 as in grades 1 (FIGO 1A, G1, and G2)?	79.0%	Round 2
15. Should a closing hysterectomy be performed if no pregnancy has been obtained at two years?	87.2%	Round 1
Surgery of early stages		
16. What treatment is recommended for an inoperable low-risk patient?	Curietherapy and external radiotherapy: 61.1%	Round 2
Assessment of lymph node status		
17. Is there a minimum number of sentinel node procedures required to operate unsupervised?	20:84.6%	Round 1
18. Should Indocyanine Green be used as the first choice, even off-label?	84.6%	Round 1
19. Should a uterine manipulator with cannulation be used?	34.5%	Round 1
20. Should the sentinel node be done in the absence of myometrial infiltration suspected on imaging?	80.7%	Round 1
21. Should the Persson algorithm be respected?	80.8%	Round 1
22. Should restaging by lumbo-aortic lymphonodectomy be systematically performed if the sentinel lymph node is positive for early stages?	60.0%	Round 2
23. Is ultrastaging recommended in the pathological analysis of the sentinel lymph node?	89.4%	Round 2
24. Is there a benefit of sentinel biopsy with frozen section in high-risk populations?	69.2%	Round 1
Intermediate risk		
25. Can brachytherapy be omitted in patients < 60 years of age?	66.6%	Round 1
26. Can brachytherapy be omitted in patients with Stage IA endometrial histology + high grade + absence of vascular emboli or focal presence of vascular emboli + p53 mutation without myometrial invasion or if limited to a polyp +?	33.3%	Round 1
27. Can brachytherapy be omitted in non-endometrial Stage IA (i.e., serous type, clear cell, undifferentiated carcinoma, carcinosarcoma, mixed) without myometrial invasion?	33.3%	Round 1
28. Should chemotherapy be given in cases of stage IA, type 2 and/or mutated p53 without myometrial invasion?	33.3%	Round 1
Intermediate–high risk		
29. What is the appropriate dose for pelvic radiotherapy in endometrial cancer?	45 Gy: 100.0%	Round 1
Intermediate–high risk (pN0 after lymph node staging)		
30. In N0 cases with emboli and/or stage II without associated lymph node involvement, is there an indication for pelvic radiotherapy in addition to brachytherapy?	88.9%	Round 1
31. In N0, is there an indication for adjuvant chemotherapy for emboli and/or high grade?	84.6%	Round 1
32. In high risk N0 is there an indication for no further treatment?	22.5%	Round 1
Intermediate–high risk (cN0/Nx)		
33. In case of incomplete surgery, is there a place for restaging in high intermediate risk patients?	94.8%	Round 1
34. In high risk cN0/Nx, is external radiation therapy to the proximal primary iliac part indicated only for stage II?	92.4%	Round 1
35. Is there an indication for adjuvant treatment with brachytherapy alone for high-grade endometrioid carcinoma stage IB without emboli or for endometrioid carcinoma grade 1-stage II?	55.5%	Round 1
High risk		
36. What is the best chemotherapy/radiation sequence for adjuvant treatment?	PORTEC: 66.7%	Round 2
37. Should the high-risk group treatment be strictly applied for p53 mutated stage I (except stage IA, see above)?	86.8%	Round 1
38. Can we do an exclusive sequential treatment (without concomitant chemotherapy)?	85.8%	Round 1
39. In high-risk endometrial cancers, can exclusive chemotherapy be used?	39.0%	Round 2
40. Should adjuvant hormone therapy be used?	39.0%	Round 1
41. Should brachytherapy be performed in addition to external radiotherapy?	95.3%	Round 1
42. Should carcinosarcomas be treated at any stage as high-risk tumours?	90.2%	Round 1
43. In the absence of recommendations on “mixed” carcinomas, should management be based on the most pejorative or most represented component?	Most pejorative: 93.6%	Round 1
44. Is chemotherapy currently required for patients with POLE mutation stage III?	66.8%	Round 2
45. Is chemotherapy currently required for patients with POLE mutation stage IV?	88.9%	Round 2
Locally advanced/metastatic tumours		
46. Should the molecular status of the tumour of metastatic patients be characterized to define chemosensitivity?	80.8%	Round 1
47. Should the molecular status of the tumour of metastatic patients be characterized to clarify monitoring and adaptation of the treatment protocol?	100.0%	Round 1
48. Should metastatic patients be routinely tested for HER2?	72.3%	Round 2
49. Should we search for PDL-1?	35.0%	Round 1
50. In case of chemotherapy/radiotherapy: Should concomitant treatment be done?	54.5%	Round 1
51. In case of chemotherapy/radiotherapy: Should we do a sequential treatment?	90.9%	Round 1
52. Should we do a surgical management of closure if there is a good response to chemotherapy and no comorbidity for surgery?	92.3%	Round 1
53. In endometroid cancers, what chemotherapy protocols do you use after radiotherapy?	4 courses with carboplatin-taxol: 44.4%	Round 2
54. In clear cell cancers what chemotherapy protocols do you do after radiotherapy?	4 courses with carboplatine-taxol: 50.0%	Round 2
55. In serous endometrial cancers, what chemotherapy protocols do you use after radiotherapy?	6 courses with carboplatin-taxol: 72.2%	Round 2
56. In carcinosarcoma, what chemotherapy protocols do you use after radiotherapy?	6 courses with carboplatine-taxol: 66.7%	Round 2

## Data Availability

Corresponding authors can show the data in case of request.
